# Integrating On-Treatment Modified Glasgow Prognostic Score and Imaging to Predict Response and Outcomes in Metastatic Renal Cell Carcinoma

**DOI:** 10.1001/jamaoncol.2023.1822

**Published:** 2023-06-22

**Authors:** Jonas Saal, Tobias Bald, Markus Eckstein, Damian J. Ralser, Manuel Ritter, Peter Brossart, Viktor Grünwald, Michael Hölzel, Jörg Ellinger, Niklas Klümper

**Affiliations:** 1 Medical Clinic III for Oncology, Hematology, Immune-Oncology, and Rheumatology, University Hospital Bonn, Bonn, Germany; 2 Institute of Experimental Oncology, University Hospital Bonn, Bonn, Germany; 3 Center for Integrated Oncology Aachen/Bonn/Cologne/Düsseldorf, Bonn, Germany; 4 Institute of Pathology, University Hospital Erlangen, Friedrich-Alexander-Universität Erlangen-Nürnberg, Erlangen, Germany; 5 Comprehensive Cancer Center EMN, University Hospital Erlangen, Friedrich-Alexander-Universität Erlangen-Nürnberg, Erlangen, Germany; 6 Department of Gynaecology and Gynaecological Oncology, University Hospital Bonn, Bonn, Germany; 7 Department of Urology and Pediatric Urology, University Hospital Bonn, Bonn, Germany; 8 Clinic for Medical Oncology, University Hospital Essen, Essen, Germany; 9 Clinic for Urology, University Hospital Essen, Essen, Germany

## Abstract

**Importance:**

In the era of immuno-oncology, imaging alone seems to be insufficient to capture treatment responses, as patients with stable disease treated with immunotherapy have a wide range of clinical outcomes. There is an unmet need for complementary (ideally cost-efficient) markers that enable assessment of therapy response and outcomes in conjunction with imaging.

**Objectives:**

To examine whether longitudinal changes in the modified Glasgow prognostic score (mGPS), which is based on C-reactive protein and albumin, can predict responses and outcomes in patients with metastatic renal cell carcinoma (mRCC).

**Design, Setting, and Participants:**

This post hoc analysis, conducted from October 2022 to April 2023, evaluated the prognostic and predictive performance of on-treatment mGPS in patients with mRCC being treated with atezolizumab (plus bevacizumab) or sunitinib in 2 randomized clinical trials: the phase 3 IMmotion151 study (discovery cohort) and the phase 2 IMmotion150 study (validation cohort).

**Main Outcomes and Measures:**

Outcomes were investigator-assessed progression-free survival (PFS) per Response Evaluation Criteria in Solid Tumors (RECIST), version 1.1 and overall survival (OS) for survival analyses. To compare the prognostic value of the on-treatment mGPS with radiologic staging, we used RECIST assessed by the Independent Review Committee (IRC-RECIST) to ensure high data quality.

**Results:**

Of the 915 patients with mRCC in the IMmotion151 discovery cohort, baseline mGPS was available for 861 patients and on-treatment mGPS for 691. The IMmotion150 validation cohort included 305 patients with mRCC, and on-treatment mGPS could be evaluated for 199. In the IMmotion150 study, on-treatment mGPS predicted outcomes as early as 6 weeks following therapy initiation, thereby opening a window for early therapy adjustments. In both clinical trials, on-treatment mGPS provided valuable prognostic information regardless of imaging-assessed treatment response at first staging. Of note, in the disease control subgroup, on-treatment mGPS exhibited superior and independent prognostic information compared with IRC-RECIST (available for 611 patients; C-index, 0.651 [95% CI, 0.588-0.714] for the mGPS during treatment vs 0.574 [95% CI, 0.528-0.619] for IRC-RECIST).

**Conclusions and Relevance:**

These data support the concept of integrating on-treatment mGPS for more holistic and patient-centered therapy monitoring in addition to radiologic staging to improve clinical care at a low cost for patients with mRCC.

## Key Points

**Question:**

Can longitudinal changes in systemic inflammatory response, measured by C-reactive protein and albumin within the modified Glasgow prognostic score (mGPS), predict response and outcomes in patients with metastatic renal cell carcinoma?

**Findings:**

In this prognostic study, which was a post hoc analysis of the IMmotion150 trial (915 patients) and IMmotion151 trial (305 patients), longitudinal measurement mGPS during treatment provided valuable prognostic information regardless of imaging-assessed treatment response. In the disease control subgroup, on-treatment mGPS exhibited superior and independent prognostic information compared with radiologic staging alone.

**Meaning:**

These findings suggest that integrating on-treatment mGPS for more holistic and patient-centered therapy monitoring in addition to radiologic staging may improve clinical care at a low cost for patients with metastatic renal cell carcinoma.

## Teaser

This prognostic study assesses the prognostic and predictive value of the modified Glasgow prognostic score during treatment in patients with metastatic renal cell carcinoma.

## Introduction

Reliable prediction of treatment responses is crucial for optimal patient care during treatment. However, in daily clinical practice, we almost exclusively rely on imaging-based staging with estimation of the tumor volume. Particularly in the era of immuno-oncology, imaging-only staging has several limitations, especially in the patient subset with stable disease (SD) while taking immune checkpoint inhibitors (ICIs), which encompasses a wide range of clinical outcomes across entities.^1^ In this context, there is an unmet need for complementary markers that enable real-time assessment of therapy response and outcomes in conjunction with imaging.

In the phase 3 IMmotion151 trial comparing atezolizumab plus bevacizumab with sunitinib in patients with metastatic renal cell carcinoma (mRCC), the high prognostic value of the modified Glasgow prognostic score (mGPS) has been highlighted.^2^ The mGPS is a simple score based on only 2 markers, serum C-reactive protein (CRP) and albumin.^2^ Of particular note, the easy-to-implement mGPS can predict outcomes as accurately as the International Metastatic Renal Cell Carcinoma Database Consortium (IMDC) score at baseline,^2^ which is the current clinical standard for risk stratification and influences treatment decisions.^3,4^

The mGPS is a well-established and robust measure for cancer-related inflammation and cachexia, both of which contribute significantly to functional decline and death.^5,6,7^ International experts have recognized the challenge of defining and refining tools, such as the mGPS, that capture the impact of systemic inflammatory processes associated with advanced cancer to enable reliable prediction of disease and treatment outcomes.^8^

Longitudinal measurement of early on-treatment inflammatory biomarkers can provide valuable information on treatment response for patients with mRCC.^9,10,11^ Whether the kinetics of mGPS measured longitudinally during treatment, which provides valuable prognostic information at baseline,^2^ predict response to treatment and can improve imaging-only therapy monitoring has not been systematically investigated. We have thus assessed the prognostic and predictive value of on-treatment mGPS in patients with mRCC treated with atezolizumab (plus bevacizumab) or sunitinib in 2 independent clinical trials, namely the phase 3 IMmotion151 study (discovery cohort) and phase 2 IMmotion150 (validation cohort).^12,13^

## Methods

This prognostic study is a post hoc analysis of data from the randomized phase 3 IMmotion151 (NCT02420821) and phase 2 IMmotion150 (NCT01984242) trials, which were provided via vivli.org.^12,13^ The trial protocols and CONSORT flow diagrams of both trials have been previously published.^12,13^ Vivli’s independent review panel, which includes an ethics branch, approved our study. This study followed the Transparent Reporting of a Multivariable Prediction Model for Individual Prognosis or Diagnosis (TRIPOD) reporting guideline.

### Estimation of mGPS at Baseline and During Treatment

The mGPS was calculated using the laboratory parameters obtained in the study. The mGPS is determined by assigning 1 point for an elevated serum CRP concentration (>10 mg/L) and, only in patients with elevated CRP levels, 1 point for a decreased serum albumin (<35 g/L).^2^ Patients are then classified as low risk (0 points), intermediate risk (1 point), and high risk (2 points).

For baseline mGPS, the laboratory parameters of the screening visit (median [IQR], 12 [7-17] days before the start of treatment) were used.^2^ To determine on-treatment mGPS, CRP levels were available for 691 patients in the IMmotion151 discovery cohort at the time of first radiologic staging (median [IQR], 85 [84-86] days for mGPS; median [IQR], 84 [81-87] days for imaging) (baseline characteristics in eTable 1 in Supplement 1; flowchart in eFigure 1 in Supplement 1). In 158 cases (21.6%; only in the IMmotion151 trial), albumin was not measured on exactly the same day as CRP; the most proximate albumin value was chosen for the assessment of on-treatment GPS, with a maximum time difference of 14 days between the evaluation of CRP and albumin accepted (median [IQR] time difference, 0 [0-0] days). In the IMmotion150 validation cohort, the first CRP value was collected approximately 6 weeks earlier according to the study protocol (median [IQR], 43 [42-43] days).

### Statistical Analysis

The IMmotion150 and IMmotion151 trials were conducted from May 2015 to February 2020, and this post hoc analysis was conducted between October 2022 and April 2023. In line with the primary efficacy objective of the phase 3 IMmotion151 trial, we used the investigator-assessed progression-free survival (PFS) per Response Evaluation Criteria in Solid Tumors (RECIST), version 1.1 and overall survival (OS) for survival analyses. To compare the prognostic value of the on-treatment mGPS with radiologic staging, we used RECIST assessed by the Independent Review Committee (IRC-RECIST) to ensure high data quality. The IRC-RECIST was available for 611 patients (eFigure 1 in Supplement 1).

Outcomes were evaluated by univariate Kaplan-Meier regression and tested with the log-rank test. Univariate and multivariate Cox proportional hazards regression analyses were performed to compare the prognostic value of mGPS with IRC-RECIST with regard to PFS and OS. The concordance index (C-index) is calculated for Cox proportional hazards regression and reported with 95% CIs. The Pearson χ^2^ test was applied to perform intergroup comparisons. All analyses were performed in R studio, version 1.4 (R Foundation for Statistical Computing) in the vivli.org secure research environment, which is a remote desktop tool. According to the Vivli guidelines, only research results and not patient-level data are allowed to be exported from the environment. Therefore, we do not have copies of the patient-level data sets.

Statistical analyses were performed using R’s survival, survminer, and rstatix packages. Figures were generated using the ggplot2 and Gmisc packages. For all statistical tests, a 2-sided *P* < .05 was considered statistically significant.

## Results

### Treatment Response and Outcomes in the Phase 3 IMmotion151 Discovery Cohort

Of the 915 patients with mRCC in the IMmotion151 discovery cohort, baseline mGPS was available for 861 patients and on-treatment mGPS for 691 (eFigure 1 in Supplement 1). Of note, 17 of 81 patients (21.0%) with high-risk mGPS at baseline died before the initial radiologic staging and were thus unavailable for on-treatment mGPS analysis, compared with only 4 of 94 (0.8%) in the low-risk mGPS group and 20 of 286 (7.0%) of the intermediate-risk mGPS group, which again highlights the high prognostic value of baseline mGPS.^1^ To explore whether reevaluation of mGPS during treatment provides additional prognostic information, we calculated on-treatment mGPS at the time of the first radiologic restaging following therapy initiation within the IMmotion151 discovery cohort.

**Figure 1.**
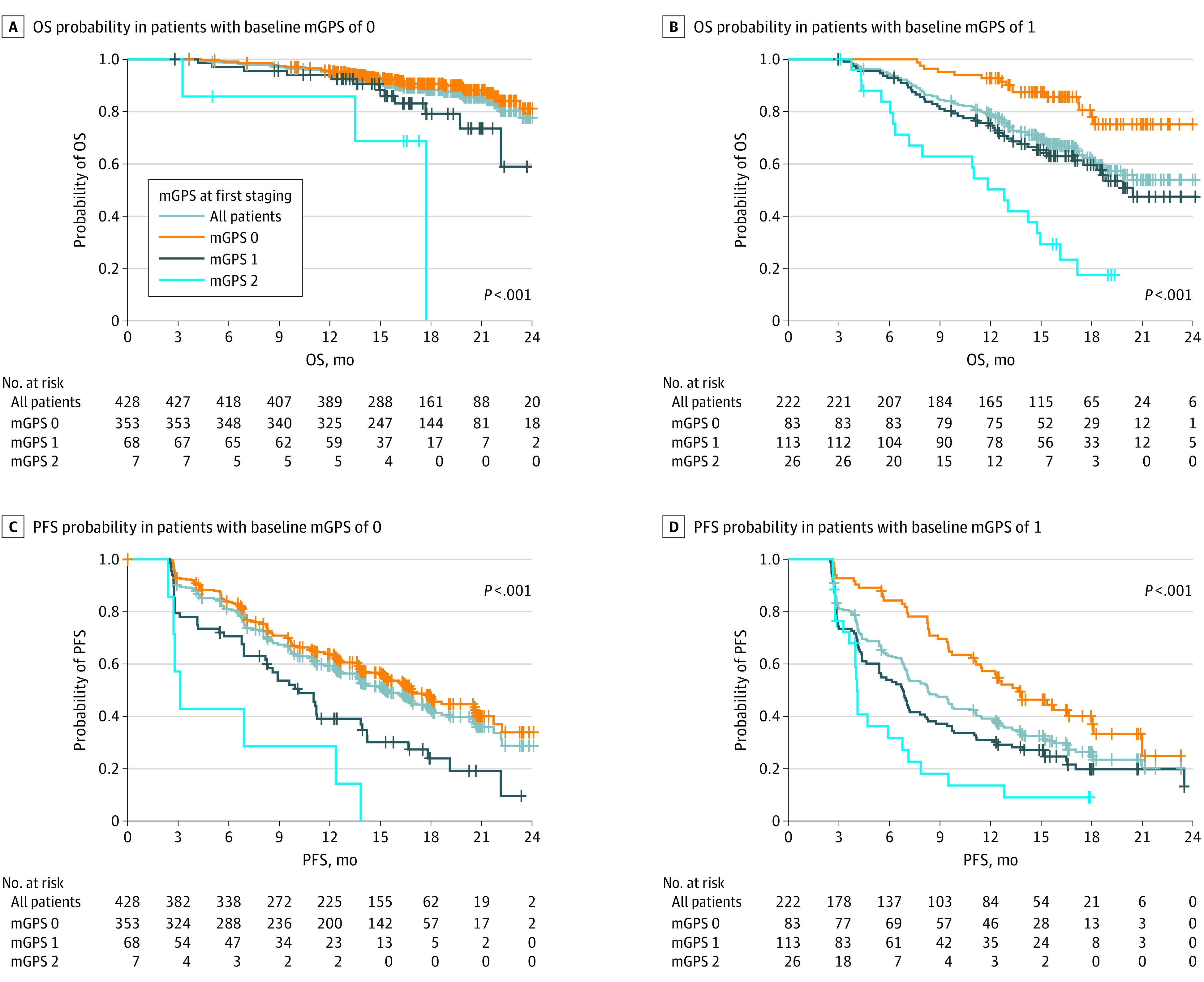
Outcome Predictions of the On-Treatment Modified Glasgow Prognostic Score (mGPS) in the IMmotion151 Discovery Cohort On-treatment mGPS in the subgroups with baseline mGPS low (mGPS 0) and intermediate (mGPS 1) risk provides prognostic information regarding both overall survival (OS) and progression-free survival (PFS). In the high-risk subgroup (mGPS 2), the on-treatment mGPS is depicted in eFigure 2 in Supplement 1. For reference, in the present Figure, the Kaplan-Meier curve of the entire baseline mGPS subgroup (all patients) is shown in each panel.

Because of the prognostic significance of baseline mGPS, on-treatment mGPS kinetics were stratified by baseline subgroups. The on-treatment mGPS provides significant prognostic information for the mGPS low- and intermediate-risk subgroups (Figure 1A and B). For example, of 222 patients with an intermediate-risk mGPS at baseline, 83 (37.4%) improved to on-treatment mGPS low-risk and had a 12-month OS of 92.8%. In comparison, 113 (50.9%) in the intermediate-risk mGPS group remained at intermediate risk and had a 12-month OS of 74.7%, and 26 (11.7%) deteriorated to high-risk mGPS with a limited 12-month OS of only 50.3%. Furthermore, patients who were in the intermediate-risk mGPS group at baseline and deteriorated to high risk had a 12-month PFS of only 13.6%, whereas improvement to low risk resulted in an approximately 4-fold higher 12-month PFS rate of 57.3%. There was a similar trend without reaching statistical significance for the high-risk mGPS group, which consists of only 41 patients (eFigure 2 in Supplement 1). Of note, on-treatment mGPS predicted treatment response and failure in all baseline mGPS subgroups (eFigure 3 in Supplement 1). Thus, the data in the IMmotion151 discovery cohort show that the changes in baseline mGPS within the first 3 months of treatment provided valuable prognostic and predictive information in addition to the already strongly prognostic baseline mGPS.^2^

### Prognostic Information in the Disease Control Group of the Phase 3 IMmotion151 Discovery Cohort

**Figure 2.**
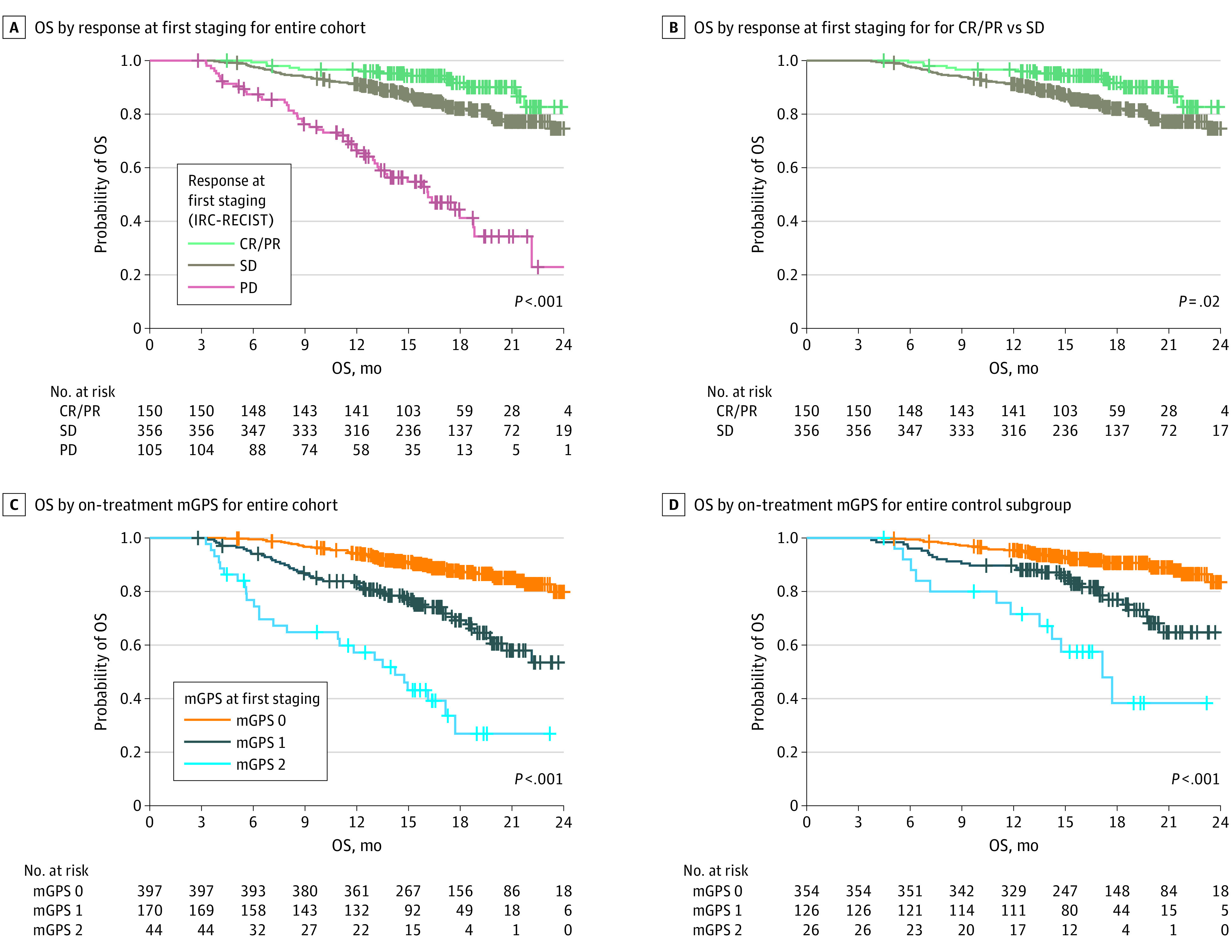
Prognostic Information of the On-Treatment Modified Glasgow Prognostic Score (mGPS) in the Disease Control (DC) Subgroup of the Phase 3 IMmotion151 Trial Progressive disease (PD) in the first staging according to the Response Evaluation Criteria in Solid Tumors assessed by the Independent Review Committee (IRC-RECIST) is associated with unfavorable overall survival (OS), whereas outcomes in the complete or partial remission (CR/PR) and stable disease (SD) subgroups differs only slightly. The on-treatment mGPS at first staging has a strong prognostic value, even in the DC subgroup (ie, patients with CR/PR or SD) and identifies a high-risk group of patients (mGPS 2) in the DC subgroup.

A well-documented limitation of radiologic staging is the low prognostic value of the differentiation between SD and partial remission (PR).^1^ This limitation was also reflected in the IMmotion151 discovery cohort, in which patients with SD demonstrated a comparable 12-month OS of 91.2% to patients with therapy response (complete remission [CR] or PR) and 12-month OS of 96.6% (Figure 2A). The small subgroup of patients with progressive disease (PD) at first staging (105 [17.2%]) exhibited a 12-month OS of only 66.5% (hazard ratio [HR] for death for PD vs complete or partial remission (CR/PR), 9.21; 95% CI, 5.00-17.00; *P* < .001) (eTable 2 in Supplement 1). Of note, on-treatment mGPS provides additional prognostic information to initial staging regardless of treatment response at first staging (eTable 3 in Supplement 1).

Because only minor prognostic information is available at the initial staging in the disease control (DC) group (SD, PR, and CR), which comprises more than 80% of the IMmotion151 cohort, we investigated whether on-treatment mGPS can provide additive prognostic information. Therefore, the prognostic value of the on-treatment mGPS assessed at the time of first staging and beyond was analyzed. In the entire IMmotion151 cohort, on-treatment mGPS showed prognostic value with a similar C-index for OS (0.685; 95% CI, 0.638-0.732) as IRC-RECIST at first staging (0.690; 95% CI, 0.647-0.733). Throughout further treatment, the mGPS still provided significant prognostic information at weeks 12, 24, and 35, underscoring the robustness of this prognostic score (eFigure 4 in Supplement 1).

In the DC subgroup, on-treatment mGPS exhibited superior prognostic information vs IRC-RECIST (HR for death, 2.71 [95% CI, 1.65-4.45] for the intermediate-risk mGPS group and 7.54 [95% CI, 3.88-14.7] for the high-risk mGPS group compared with the low-risk mGPS group [*P* < .001 for both] vs 2.05 [95% CI, 1.13-3.74; *P* = .01] for the SD subgroup compared with the PR/CR subgroup by IRC-RECIST; C-index, 0.651 [95% CI, 0.588-0.714] for the on-treatment mGPS vs 0.574 [95% CI, 0.528-0.619] for IRC-RECIST) (Table). Furthermore, in multivariate Cox proportional hazards regression, on-treatment mGPS showed strong and independent prognostic value, whereas the IRC-RECIST did not robustly predict outcome in the DC group (HR, 1.63; 95% CI, 0.89-3.00; *P* = .11 for SD vs CR/PR) (Table).

**Table.** Univariate and Multivariate Cox Proportional Hazards Regression Analysis for mGPS and IRC-RECIST at First Staging for the 506 Patients in the Disease Control Subgroup of the IMmotion151 Cohort

Characteristic	Overall survival		Progression-free survival	
	HR (95% CI)	*P* value	HR (95% CI)	*P* value
**Univariate analysis**				
mGPS at first staging				
0	1 [Reference]	NA	1 [Reference]	NA
1	2.71 (1.65-4.45)	<.001	1.69 (1.30-2.19)	<.001
2	7.54 (3.88-14.7)	<.001	3.24 (2.03-5.17)	<.001
Response in first staging (IRC-RECIST)				
CR/PR	1 [Reference]	NA	1 [Reference]	NA
SD	2.05 (1.13-3.74)	.02	1.38 (1.05-1.80)	.02
**Multivariate analysis**				
mGPS at first staging				
0	1 [Reference]	NA	1 [Reference]	NA
1	2.56 (1.55-4.22)	<.001	1.64 (1.26-2.14)	<.001
2	6.83 (3.48-13.4)	<.001	3.05 (1.90-4.90)	<.001
Response in first staging (IRC-RECIST)				
CR/PR	1 [Reference]	NA	1 [Reference]	NA
SD	1.63 (0.89-3.00)	.11	1.23 (0.94-1.62)	.14

Abbreviations: CR, complete remission; HR, hazard ratio; IRC-RECIST, Independent Review Committee Response Evaluation Criteria in Solid Tumors; mGPS, modified Glasgow prognostic score; NA, not applicable; PR, partial response; SD, stable disease.

Importantly, on-treatment mGPS allowed for the identification of a high-risk group within the DC subgroup that has comparable OS to patients with PD at initial staging (median OS, 17.1 vs 16.1 months; 12-month OS, 71.6% vs 66.5%) (Figure 2). Even though this subgroup was small (26 patients [5.1%] with DC), our findings suggest that those patients should be closely monitored. Our data therefore strongly suggest that in the DC group, which represents a substantial subgroup of patients with mRCC in the first line (DC group >80% in IMmotion151), the simple and inexpensive determination of on-treatment mGPS provides valuable additional prognostic information to radiologic staging.

### Early Prognostic Information in the Phase 2 IMmotion150 Validation Cohort

**Figure 3.**
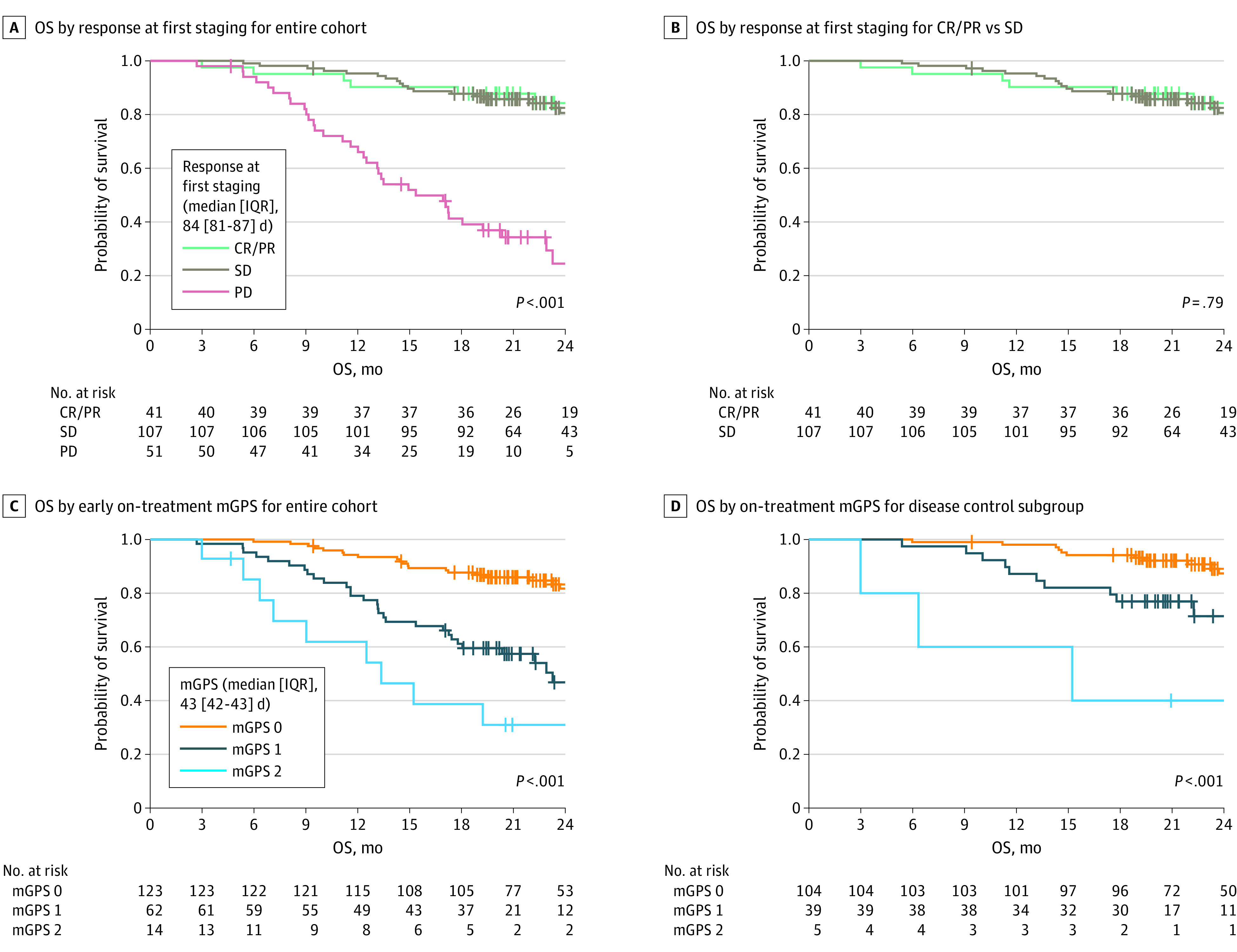
Outcome Predictions as Early as 6 Weeks After Therapy Start With the Use of the On-Treatment Modified Glasgow Prognostic Score (mGPS) Patients with progressive disease (PD) according to the Response Evaluation Criteria in Solid Tumors assessed by the Independent Review Committee (IRC-RECIST) have unfavorable overall survival (OS), whereas OS is indistinguishable in the stable disease (SD) and complete remission or partial remission (CR/PR) subgroups. The on-treatment mGPS measured 6 weeks after the start of treatment accurately predicts OS in the entire cohort and disease control (DC) subgroup.

To validate the data from the IMmotion151 discovery cohort, we used the similarly designed phase 2 IMmotion150 study. In this trial, 305 patients with mRCC were treated with atezolizumab, sunitinib, or a combination of atezolizumab and bevacizumab in the first-line setting. For 199 patients, on-treatment mGPS could be evaluated. Importantly, in this study, the on-treatment mGPS could be determined at week 6 (median [IQR], 43 [42-43] days) and thus approximately 6 weeks earlier than in IMmotion151 (median [IQR], 85 [84-86] days). Thus, in addition to confirming our results from the IMmotion151 cohort, we were able to demonstrate that on-treatment mGPS allowed outcome prediction as early as 6 weeks (median, 43 days) after therapy initiation, which is a substantial time before the first radiologic staging (median, 85 days) (Figure 3). Because the first routine staging is usually performed 8 to 12 weeks after the start of therapy, early on-treatment mGPS could open a potential window for early therapy adjustments.

## Discussion

In this study, we highlight the prognostic and predictive value of on-treatment mGPS. Importantly, on-treatment mGPS provides valuable prognostic information, particularly in the large DC group (>80%), where standard imaging has only limited discriminatory power. We validate our findings in 2 independent clinical trials (IMmotion150 and 151) in patients with mRCC treated in the first-line setting. Furthermore, we demonstrate that on-treatment mGPS had a high prognostic value as early as 6 weeks after treatment initiation, thus providing valuable information well before radiologic staging, which is typically initiated after 12 weeks. Hence, assessment of early on-treatment mGPS might allow physicians to identify patients at highest risk for progression to adjust their treatment accordingly or to initiate earlier radiologic response evaluation.

The mGPS is a simple score, consisting of 2 routine laboratory parameters. Hence, it can be measured cost-effectively in any clinical laboratory and can be easily implemented into everyday clinical practice, even in low-resource settings. Our results therefore provide a strong rationale to integrate on-treatment mGPS into routine clinical practice for improved patient monitoring. Furthermore, because CRP is currently not routinely determined at baseline in most large randomized trials, we propose that the measurement of CRP at baseline and early CRP during treatment should be included in future clinical trial protocols to further validate the use of on-treatment markers in different treatments and patient populations.

Research has demonstrated a correlation of mGPS with survival across several tumor entities at baseline.^5,7^ Whether on-treatment mGPS also has predictive value in other cancer types remains to be elucidated. In the first-line mRCC treatment setting, baseline mGPS has been shown to predict outcomes as accurately as the IMDC score, which is the current clinical standard for risk stratification: The C-index for baseline mGPS was 0.71 (95% CI, 0.68-0.74) compared with the IMDC score 0.63 (95% CI, 0.60-0.66).^2^ Because the IMDC score includes clinical items, such as “time from diagnosis to initiation of systemic therapy (12 months),” it cannot be determined longitudinally during therapy, unlike the mGPS, which integrates only laboratory parameters. Our data on the prognostic and predictive power of mGPS measured at baseline and during treatment strongly support the integration of mGPS for risk stratification for response and outcome prediction.

Another prominent example of an inflammatory on-treatment biomarker that can predict ICI response is early CRP kinetics. Studies^9,10,14^ have shown that the so-called CRP flare-response phenomenon predicts immunotherapy response in diverse entities, including mRCC. Patients with a CRP flare response, characterized by an early increase in CRP within the first month of therapy and a subsequent decrease to baseline within 3 months, demonstrated exceptional long-term survival when undergoing immunotherapy. In patients with non–small cell lung cancer, CRP flare-response kinetics already occur in a substantial proportion of patients within a period of only 4 to 6 weeks.^15^ In the IMmotion150 study, on-treatment mGPS provides valuable prognostic information as early as 6 weeks after therapy initiation. We hypothesize that the prognostic value of on-treatment mGPS within the first month of therapy may be perturbed by flare-like kinetics because an increasing CRP value (flare-like) would lead to an increased mGPS. Because no earlier CRP measurements are available for the IMmotion150 and 151 trials, this hypothesis remains to be confirmed.

The treatment of mRCC has developed rapidly in recent years.^3^ Currently, 2 predominant drug classes are applied to treat patients with mRCC: tyrosine kinase inhibitors (TKIs) and ICIs. Currently, 4 combination therapies considered equivalent in clinical efficacy are used in the first-line treatment of intermediate and high-risk mRCC. Essentially, these therapeutic concepts can be classified as intensified ICIs (anti–PD-1 and anti–CTLA-4) vs a combination of an ICI and antiangiogenic TKI (anti–PD-1 and vascular endothelial growth factor receptor–TKI). All current therapeutic combinations in the first-line setting for mRCC induce disease control rates higher than 80%. In the large patient subgroup with DC, radiologic staging provides minimal prognostic information,^8^ which is also underscored by our data from IMmotion150 and 151 (Figure 2 and Figure 3). In particular, the patient group with SD encompasses a variety of outcomes.^1^ However, real-time determination of benefit in patients with SD is critical to robustly inform clinical decisions, guide continuation of effective therapy, and avoid unnecessary toxic effects or costs of ineffective treatment.

The mGPS is an inflammatory score that correlates with sarcopenia^7^ and tumor cachexia.^16^ The term *laboratory cachexia* has been defined similarly to mGPS as a CRP level greater than 10 mg/L and coinciding albumin less than 35 g/L by Gray et al.^17^ Of note, the prevalence of laboratory cachexia increases markedly up to 85% 0 to 30 days before death.^17^ These data underscore the high mortality in patients with a high-risk mGPS constellation and laboratory cachexia and suggest that the mGPS is capable of measuring not only tumor dynamics but also the patient’s inflammatory and metabolic status. Thus, longitudinal assessment of the mGPS can provide a more comprehensive picture of the patient’s general condition than radiologic assessment of tumor volumes alone.

### Limitations

Despite some strengths, such as the investigation of 2 similarly designed but independent prospective clinical trials comprising a total of 890 patients (691 in the discovery cohort and 199 in the validation cohort) with available on-treatment mGPS data, our study also has some limitations. First, CRP was measured at different time points, which allowed us to investigate the value of the early on-treatment mGPS at week 6 only in the IMmotion150 trial. Furthermore, CRP and albumin are both nonspecific markers of inflammation that may be altered in a variety of conditions. This fact warrants careful investigation of confounding factors, such as infection or treatment-related inflammation, that could affect mGPS dynamics in future studies. In addition, the value of on-treatment mGPS remains to be investigated in the currently used first-line therapy combinations or the combination of an ICI plus an antiangiogenic TKI. However, in the pivotal phase 3 JAVELIN-101 (A Study of Avelumab With Axitinib Versus Sunitinib in Advanced Renal Cell Cancer) trial, patients with mRCC treated with avelumab plus axitinib who had an elevated baseline CRP level of 10 mg/L or higher that decreased at least once below 10 mg/L (termed *normalized*) within 6 weeks had superior outcomes than the nonnormalized group.^11^ Because Tomita et al^11^ used the same CRP threshold that is used for mGPS assessment (low-risk mGPS vs intermediate- or high-risk mGPS), our results may also apply to ICI and TKI combination therapy. However, to definitively establish the role of on-treatment inflammatory biomarkers, such as (on-treatment) mGPS, for patients with mRCC or other cancer types, large randomized trials need to include them in their study design.

## Conclusions

This prognostic study supports the new concept of integrating on-treatment mGPS for more holistic and patient-centered therapy monitoring in addition to radiologic staging to improve clinical care at a low cost for patients with mRCC.

## Notes

Supplement 1. **eFigure 1.** Flowchart Showing the Number of Patients Included for Our Analyses **eFigure 2.** On-Treatment mGPS in the High-Risk Subgroup **eFigure 3.** The On-Treatment mGPS Predicts Treatment Response in the IMmotion151 Discovery Cohort **eFigure 4.** In the Phase 3 IMmotion151 Trial, On-Treatment mGPS Predicts Outcomes at 12, 24 and 35 Weeks **eTable 1.** Baseline Characteristics in the IMmotion151 Cohort, Stratified by mGPS at First Staging **eTable 2.** Univariate and Multivariate Cox-Regression Analysis of the Entire IMmotion151 Cohort for On-Treatment mGPS and IRC-RECIST at First Staging **eTable 3.** Univariate Cox-Regression Analysis for mGPS for the IRC-RECIST Subgroups in the IMmotion151 Trial

Supplement 2.
Data Sharing Statement
