# Supplementary material for: Integrating On-Treatment Modified Glasgow Prognostic Score and Imaging to Predict Response and Outcomes in Metastatic Renal Cell Carcinoma
Source: JAMA Oncol. 2023 Jun 22;9(8):1048–55. doi: 10.1001/jamaoncol.2023.1822 (PMC10288377; doi:10.1001/jamaoncol.2023.1822)
Supplement: Supplement 1. — eFigure 1. Flowchart Showing the Number of Patients Included for Our Analyses eFigure 2. On-Treatment mGPS in the High-Risk Subgroup eFigure 3. The On-Treatment mGPS Predicts Treatment Response in the IMmotion151 Discovery Cohort eFigure 4. In the Phase 3 IMmotion151 Trial, On-Treatment mGPS Predicts Outcomes at 12, 24 and 35 Weeks eTable 1. Baseline Characteristics in the IMmotion151 Cohort, Stratified by mGPS at First Staging eTable 2. Univariate and Multivariate Cox-Regression Analysis of the Entire IMmotion151 Cohort for On-Treatment mGPS and IRC-RECIST at First Staging eTable 3. Univariate Cox-Regression Analysis for mGPS for the IRC-RECIST Subgroups in the IMmotion151 Trial [file jamaoncol-e231822-s001.pdf]

Supplementary Online Content

Saal J, Bald T, Eckstein M, et al. Integrating on-treatment modified Glasgow prognostic score and imaging to predict response and outcomes in metastatic renal cell carcinoma. *JAMA Oncol*. Published online June 22, 2023. doi:10.1001/jamaoncol.2023.1822

eFigure 1. Flowchart Showing the Number of Patients Included for Our Analyses
eFigure 2. On-Treatment mGPS in the High-Risk Subgroup
eFigure 3. The On-Treatment mGPS Predicts Treatment Response in the IMmotion151 Discovery Cohort
eFigure 4. In the Phase 3 IMmotion151 Trial, On-Treatment mGPS Predicts Outcomes at 12, 24 and 35 weeks
eTable 1. Baseline Characteristics in the IMmotion151 Cohort, Stratified by mGPS at First Staging
eTable 2. Univariate and Multivariate Cox-Regression Analysis of the Entire IMmotion151 Cohort for On-Treatment mGPS and IRC-RECIST at First Staging
eTable 3. Univariate Cox-Regression Analysis for mGPS for the IRC-RECIST Subgroups in the IMmotion151 Trial

This supplementary material has been provided by the authors to give readers additional information about their work.

© 2023 Saal J et al. *JAMA Network Open.*

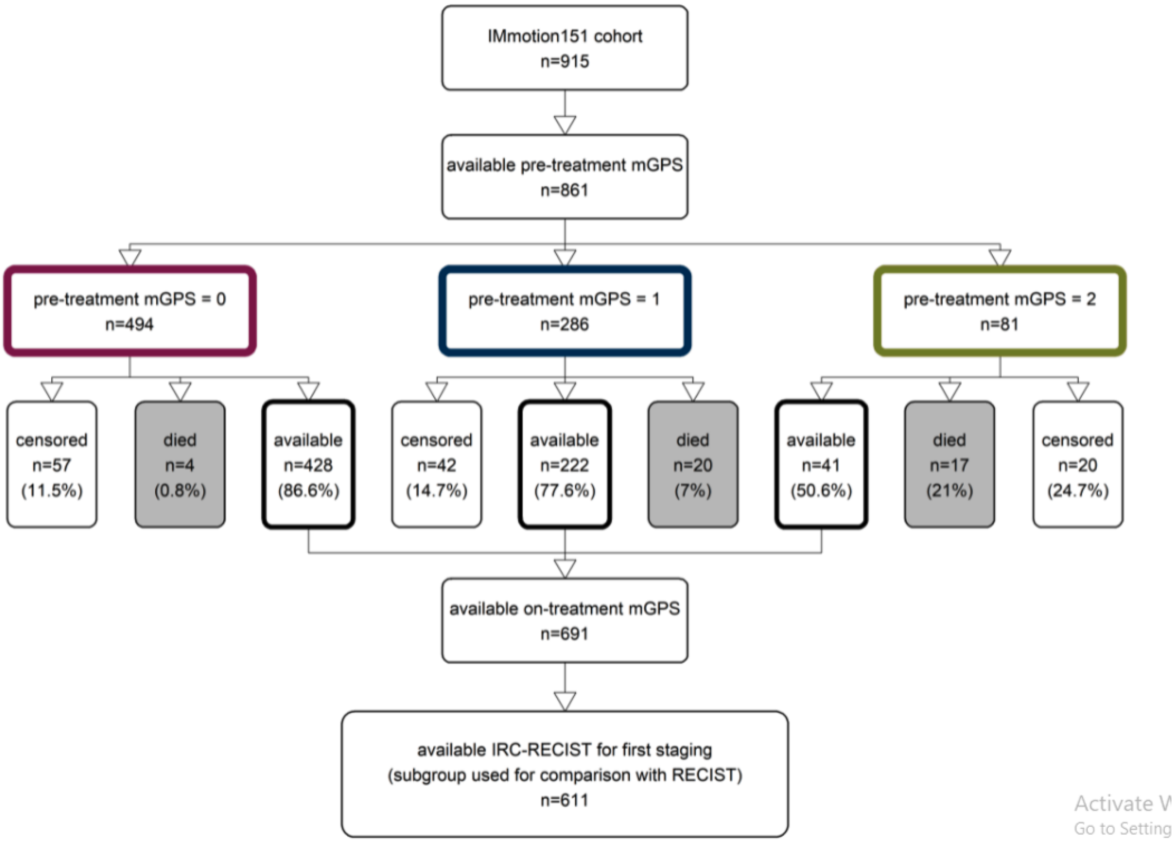

eFigure 1: Flowchart showing the number of patients included for our analyses.

© 2023 Saal J et al. *JAMA Network Open.*

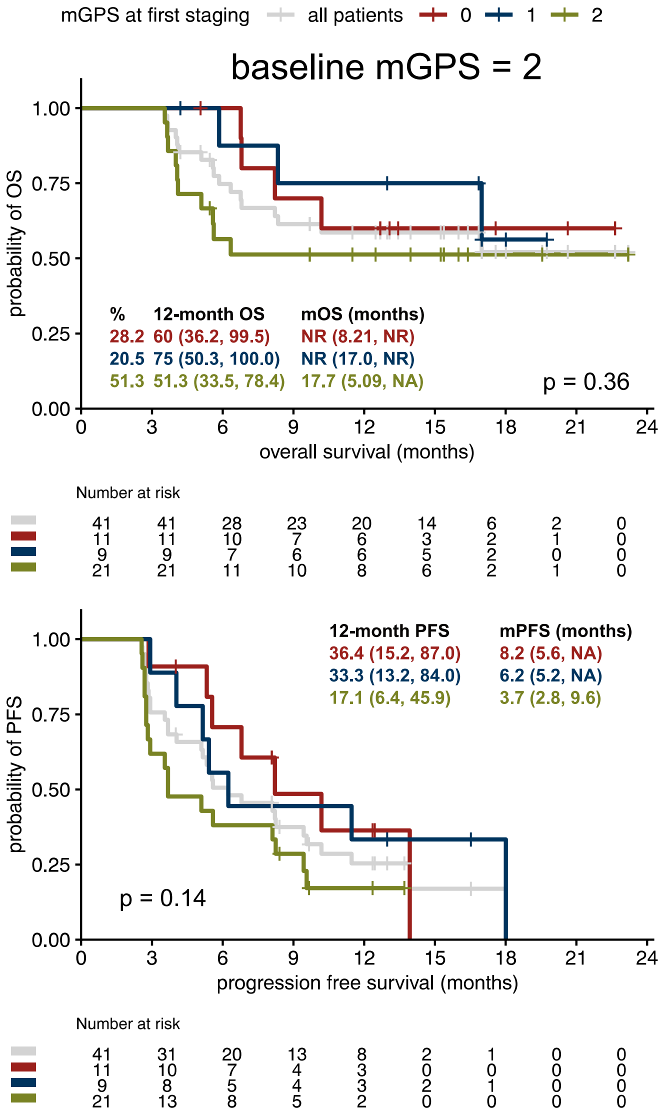

eFigure 2: On-treatment mGPS in the high-risk subgroup

© 2023 Saal J et al. *JAMA Network Open.*

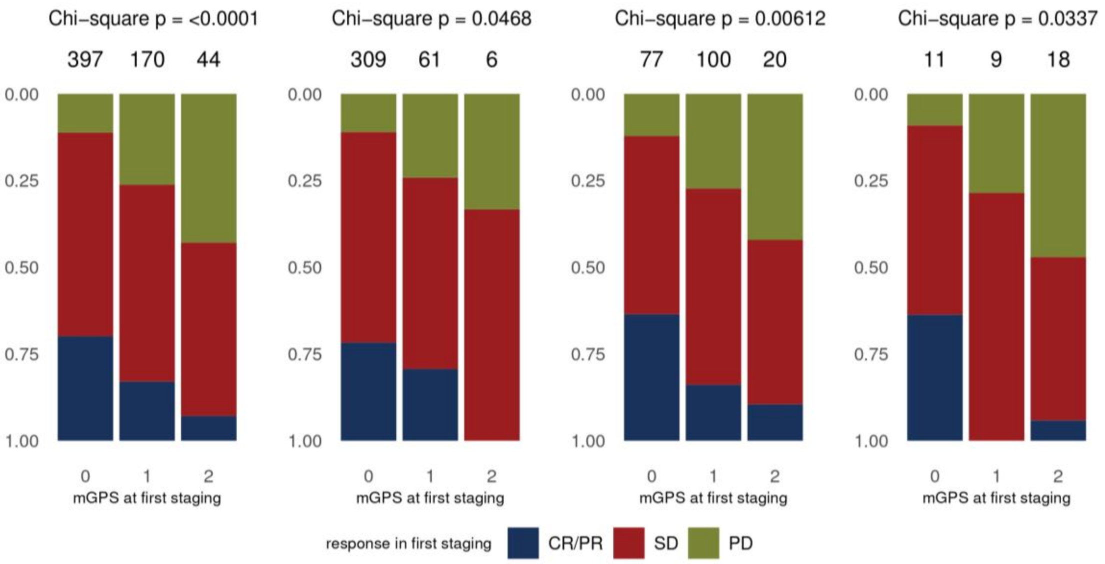

eFigure 3: The on-treatment mGPS predicts treatment response in the IMmotion151 discovery cohort. On-treatment mGPS, for the whole cohort but also the mGPS subgroups, is significantly associated with response at the time at first radiological staging as defined by IRC RECIST evaluation. Sample sizes for the mGPS subgroups are shown above the bar. PD, progressive disease; SD, stable disease; CR/PR, partial or complete remission.

© 2023 Saal J et al. *JAMA Network Open.*

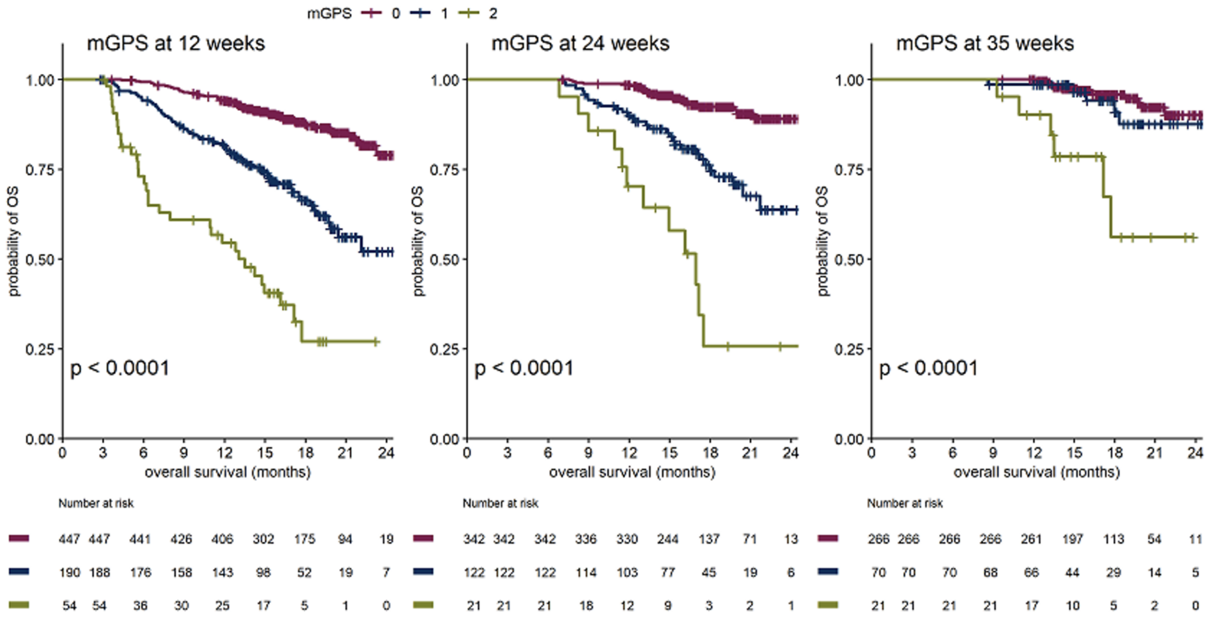

eFigure 4: In the Phase 3 IMmotion151 trial, on-treatment mGPS predicts outcomes at 12, 24 and 35 weeks.

© 2023 Saal J et al. *JAMA Network Open.*

eTable 1: Baseline characteristics in the IMmotion151 cohort, stratified by mGPS at first staging.

Characteristic	N = 691
age (years)	
< 65	443 (64%)
≥ 65	248 (36%)
sex	
female	177 (26%)
male	514 (74%)
IMDC score	
low risk	172 (25%)
intermediate risk	426 (62%)
high risk	93 (13%)
PD-L1 status (IC score ≥1)	
negative	424 (61%)
positive	267 (39%)
prior nephrectomy	520 (75%)
Karnofsky performance status	
< 80	141 (21%)
80-90	250 (36%)
100	296 (43%)
Unknown	4

© 2023 Saal J et al. *JAMA Network Open.*

eTable 2: Univariate and multivariate Cox-regression analysis of the entire IMmotion151 cohort for on-treatment mGPS and IRC-RECIST at first staging.

	OS				PFS			
**Characteristic**	**N**	**HR**[1]	**95% CI**[1]	**p-value**	**N**	**HR**[1]	**95% CI**[1]	**p-value**
univariate cox, all patients								
**mGPS at first staging**	611				611			
0		—	—			—	—	
1		2.93	1.98, 4.34	**<0.001**		1.88	1.51, 2.35	**<0.001**
2		8.88	5.48, 14.4	**<0.001**		3.83	2.70, 5.44	**<0.001**
**response in first staging (IRC)**	611				611			
CR/PR		—	—			—	—	
SD		2.06	1.13, 3.74	**0.018**		1.36	1.04, 1.79	**0.025**
PD		9.21	5.00, 17.0	**<0.001**		6.84	5.00, 9.35	**<0.001**
multivariate cox, all patients								
**response in first staging (IRC)**	611				611			
CR/PR		—	—			—	—	
SD		1.67	0.91, 3.05	0.10		1.23	0.94, 1.62	0.13
PD		6.08	3.24, 11.4	**<0.001**		5.56	4.02, 7.68	**<0.001**
**mGPS at first staging**	611				611			
0		—	—			—	—	
1		2.32	1.55, 3.46	**<0.001**		1.57	1.25, 1.98	**<0.001**
2		6.21	3.78, 10.2	**<0.001**		2.79	1.94, 4.00	**<0.001**

[1] HR = Hazard Ratio, CI = Confidence Interval

© 2023 Saal J et al. *JAMA Network Open.*

eTable 3: Univariate Cox-regression analysis for mGPS for the IRC-RECIST subgroups in the IMmotion151 trial.

	OS				PFS			
**Characteristic**	**N**	**HR**[1]	**95% CI**[1]	**p-value**	**N**	**HR**[1]	**95% CI**[1]	**p-value**
**CR/PR**								
mGPS at first staging	150				150			
0		—	—			—	—	
1		2.75	0.82, 9.25	0.10		2.17	1.27, 3.71	**0.004**
2		8.67	1.06, 71.0	**0.044**		11.1	3.36, 36.8	**<0.001**
**SD**								
mGPS at first staging	356				356			
0		—	—			—	—	
1		2.52	1.45, 4.37	**<0.001**		1.50	1.11, 2.04	**0.009**
2		6.52	3.20, 13.3	**<0.001**		2.58	1.54, 4.30	**<0.001**
**PD**								
mGPS at first staging	105				105			
0		—	—			—	—	
1		1.92	0.99, 3.72	0.052		1.38	0.88, 2.14	0.2
2		5.34	2.54, 11.2	**<0.001**		2.00	1.13, 3.54	**0.018**

[1] HR = Hazard Ratio, CI = Confidence Interval

© 2023 Saal J et al. *JAMA Network Open.*
