# Supplementary material for: Integrating On-Treatment Modified Glasgow Prognostic Score and Imaging to Predict Response and Outcomes in Metastatic Renal Cell Carcinoma
Source: JAMA Oncol. 2023 Jun 22;9(8):1048–55. doi: 10.1001/jamaoncol.2023.1822 (PMC10288377; doi:10.1001/jamaoncol.2023.1822)
Supplement: Supplement 2. — Data Sharing Statement [file jamaoncol-e231822-s002.pdf]

Data Sharing Statement

Saal. Integrating On-Treatment Modified Glasgow Prognostic Score and Imaging to Predict Response and Outcomes in Metastatic Renal Cell Carcinoma. *JAMA Oncol*. Published June 22, 2023. doi:10.1001/jamaoncol.2023.1822

Data
Data available: Yes
Data types: Participant data with identifiers
How to access data: This publication is based on research using data from Roche that has been made available through Vivli, Inc.
When available: With publication

Supporting Documents
Document types: None

Additional Information
Who can access the data: anyone requesting the data through Vivli, Inc.
Types of analyses: for any purpose
Mechanisms of data availability: after approval of a proposal and with a signed data access agreement
